# Court-ordered inpatient psychiatric care in Switzerland: determinants of length of stay and treatment outcome

**DOI:** 10.3389/fpsyt.2023.1222337

**Published:** 2023-10-03

**Authors:** Kerstin Weber, Sandrine Morier, Lise Lesaffre, Christophe Menu, Philippe Bertschy, François R. Herrmann, Panteleimon Giannakopoulos

**Affiliations:** ^1^Division of Institutional Measures, Medical Direction, Geneva University Hospitals, Geneva, Switzerland; ^2^Department of Psychiatry, Faculty of Medicine, University of Geneva, Geneva, Switzerland; ^3^Department of Institutions and Information Technology, Republic and State of Geneva, Geneva, Switzerland; ^4^Department of Rehabilitation and Geriatrics, Geneva University Hospitals and University of Geneva, Geneva, Switzerland

**Keywords:** mentally disordered offenders, court-ordered treatments, forensic psychiatry, prison, length of stay

## Abstract

**Introduction:**

In several European countries, offenders with decreased or abolished responsibility and high risk of recidivism due to long-lasting mental disorders are compulsory admitted for court-ordered treatments (COT) that take place in high and medium-security hospitals. As a rule, length of stay in these structures is very long implying major restrictions for the inmate and high societal cost. Despite intensive research, the predictors of length of stay and treatment outcome in long stay forensic services is still matter of debate.

**Methods:**

We report here a detailed analysis of the demographic, psychiatric and offense predictors of length of stay and discharge locations of 204 mentally disordered offenders convicted to COT in a new medium-security forensic psychiatry clinic in Geneva, Switzerland. Kaplan-Meier survival estimates were performed to determine time to release. Length of stay was predicted by Cox regressions, and discharge locations were predicted by multinomial logistic regressions.

**Results:**

The typical inpatient was a 35-age single male re-offender, submitted to COT after a conviction for physical violence (78.9%) or property violation (64.2%), with drug trafficking (52.9%), in relation to psychotic (67.2%), antisocial or borderline personality disorder (35.8%) with comorbid substance use disorders (60.3%). Sex offenses were found in 24.5% of cases and were associated with Cluster B personality disorders. The median length of stay was of 2.5 years and was independent of demographic variables, severity of crime recidivism and psychiatric diagnosis. Longer COT at admission, and type of offense (in particular drug traffic and sexual violence) predicted longer stays. At discharge, 32.8% of cases were transferred to sheltered educational housing, 23.1% to open low-security wards, while 30.6% returned to regular prisons and 9.7% to their country of origin.

**Discussion:**

Younger age and conviction for property violation rather than physical violence increased the chances to be discharged to sheltered educational housing. Longer COT at admission, personality disorders, and conviction for sexual offense increased the risk to return to prison. These data suggest that sex offenses determine not only longer stays under COT but also drastically decreases the chance of freedom for inmates with Cluster B personality disorders.

## Introduction

Forensic psychiatric treatment of mentally disordered offenders (MDO) has been thought to be a pragmatic and successful way of reducing criminal recidivism (1). Some European countries, such as England and Finland, regulate inpatient treatment of MDO by their mental health acts, while in other countries such as Austria, Belgium and Switzerland, the judicial framework focuses on the possibility to provide a complete panel of psychiatric care interventions in prisons (1–5).

Two main tracks of MDO can be identified. The first concerns criminally responsible offenders that receive outpatient consultations on a voluntary basis in regular prisons (6–8). When needed, inpatient mental health care for this population of inmates is usually provided in psychiatric hospitals or, more rarely, in forensic psychiatry wards (9–11). The second refers to offenders with decreased or abolished responsibility and high risk of recidivism due to long-lasting mental disorders. Several European countries rely on a medical definition of criminal irresponsibility, according to which an individual is not criminally liable when he/she, at the time of the offense, suffers from a serious mental disorder (psychiatric or neuropsychiatric disorder), that annihilates or seriously impairs his/her judgment and capacity of appreciating the illegal nature of the act, or control over his actions. Legal insanity is based on cognitive and/or volitional impairment according to countries. There is a general agreement that a diagnosis of schizophrenia indicates a lack of accountability, whereas opinions differ among legal frameworks regarding personality disorders, psychopathy, and substance use disorders (12). Current criminal justice systems shift their focus from punishment to prevention via medical treatments for these MDO (13, 14). They may be compulsory admitted for court-ordered treatments (COT) that take place in high and medium-security hospitals (11, 15, 16). COT raise ethical questions, as length of stay may be long and often indefinite (2, 4). Psychiatric care in secure prison-based settings is thus restrictive for the individual and of high cost for the society (4).

Legal frameworks governing detention vary across European countries so that the characteristics of COT are not easily comparable (1, 2, 4). Over the last 20 years, detailed description of inpatient COT was provided in Austria (17), Belgium (18), France (8, 19), Germany (20), Netherlands (21), and United Kingdom (22). The Swiss Criminal Code^1^ distinguishes between penalties and COT, named therapeutic measures. The latter are ordered when a penalty alone is not sufficient to counter the risk of future offending and the offenders requires treatment in the interest of public safety. Therapeutic measures can be pronounced in conjunction with a custodial sentence, or against offenders who are criminally irresponsible and cannot be sentenced to a penalty. The court must base its decision on a psychiatric expert assessment to provide an opinion on the prospect of success of the treatment, the probability of future offences, and the ways in which the measure may be implemented. Measures include inpatient COT of mental disorders or addictions, outpatient treatments, or lifelong imprisonment. They are reviewed regularly according to the best interest of both the individual and the public safety, because their duration can far exceed the sentence related to the seriousness of the crime, which typically determines the duration of imprisonment.

In order to improve the quality of COT and guarantee the best balance between public safety and rehabilitation of MDO, a new structure offering intensive inpatient COT from all French and Italian-speaking Swiss counties has been created in Geneva, Switzerland in 2014. This specialized medium-security forensic psychiatry clinic (referred to as “Curabilis”) is located within the central prison of the city. Its innovative go-between political and financial foundations and hybrid medical-carceral management were created to optimize the flow between the local psychiatric and correctional institutions, to ease the care-control coordination between mental health and prison professionals, and to simultaneously achieve disease recovery and criminal desistance in MDO according to European guidelines on forensic psychiatry (4). The medical responsibility in Curabilis is assumed by a newly created Division of Institutional Measures of the University Hospitals of Geneva. The strength of this approach is that the same COT can be carried out in a variety of settings along the care-control continuum, while remaining under the same judicial control and the same medical supervision. Depending on the MDOs’ degree of dangerousness and risk of recidivism, their COT can be carried out in a traditional prison ward, a specialized high-security forensic clinic such as Curabilis, a low-security psychiatric ward, or in an outpatient setting. This constellation offers an increased flexibility to minimize societal costs and liberty restriction for the individual.

Treatment in Curabilis is inspired by the forensic therapeutic community model such as described by Maxwell Jones (23–25). Each of the 5 wards hosts 15 to 18 inpatients in a discrete wing, with its own collection of cells, group, dining, and living and therapy rooms, as well as staff offices. Daily program is organized based on community meetings attended by all inmates and mental health and prison professionals, small therapy, and creative and recreational activity groups, individual psychiatric-psychotherapy sessions, and psychotropic medication, as well as prison activities such as exercise, work, and education. Timetables must fit around the times the inmates are locked up (24). In each ward, a multidisciplinary team of forensic mental health professionals (psychiatrists, psychologists, mental health nurses, movement, and occupational therapists) closely works together with prison officers, legal and social workers, chaplains, as well as education teachers and vocational trainers (leading laundry, cleaning, bakery, cooking, and gardening workshops). The forensic therapeutic community philosophy is grounded in an explicitly relational paradigm, using social skills training and interpersonal approaches, to address attachment, criminal and current behavior on the ward (26). It creates and sustains an enabling environment, where all those involved experience a sense of belonging, where rule breaking, and anti-social behaviors are challenged and explored (24, 25). Offence-paralleling behavior emerges, and the rigorous culture of enquiry allows for the acquisition of prosocial models of thinking (27).

We report here a detailed analysis of the demographic, diagnostic and criminological predictors of treatment duration and discharge locations of the first 200 MDOs admitted to this forensic psychiatry clinic. Since women represent a minority of MDO and their clinical profile remain poorly explored (28), we chose to keep a mixed sample to account for possible gender-specific differences. Based on previous data (29–33), we hypothesize that longer stays are determined mainly by a more severe criminal history (such as sexual offenses or long COTs) as well as a more severe psychiatric condition (persistent psychosis, severe personality disorders), but not by demographic characteristics. Second, we hypothesize that the majority of the MDO will be able to be discharged from prison after their specialized psychiatric forensic treatment (4). Those MDO with a more severe criminal background and a more severe psychiatric conditions would have the lowest chances to be released from prison to low-security psychiatric ward or a sheltered housing, independently of their demographic characteristics (29–33).

## Methods

### Data extraction

This study includes a range of data collected routinely. During the 9 years since its opening in 2014, 213 forensic psychiatric inmates have been consecutively admitted. Data were extracted from their psychiatric records from April 1st, 2014 to December 31st, 2022. After deduction of the 9 inmates who have refused to give informed consent, the final sample of this single episode-related study includes 204 participants under COT.

The data collected include demographic characteristics (gender, age, nationality, level of formal education, marital status, number of children), criminal offenses (type of offense, previous convictions), psychiatric diagnosis at the origin of the COT, time since the initial Court order, and treatment pathways (referral, discharge location, length of stay). Psychiatric diagnosis according to ICD-10 criteria (34) were extracted from the psychiatric expert assessments. All these diagnoses were confirmed by two independent fully trained psychiatrists at admission in Curabilis.

### Outcomes

**Figure 1:**
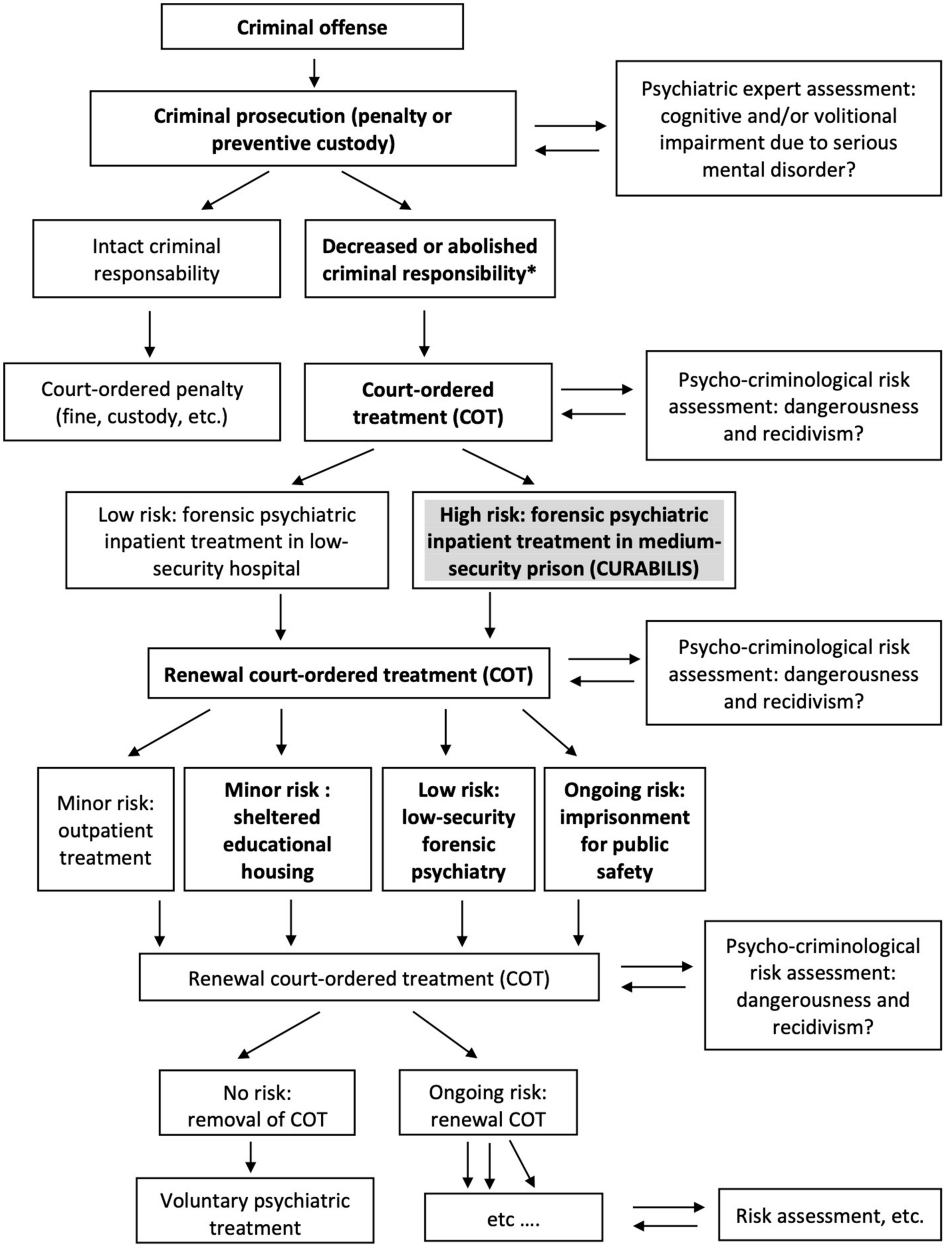
Flow chart of the Swiss Justice System. (* in bold = pathway of study participants).

We further assessed two outcomes in this study. First, length of stay is defined by the duration between the date of entry and release from Curabilis as decided by the court. As illustrated in the flowchart (Figure 1), the Swiss Justice System is based on a close collaboration and constant step-by-step interaction between the Court, forensic psychiatric experts, and psycho-criminologists. Inpatient COT in Curabilis is not limited in time but assessed annually by an *ad hoc* Court based on the continuous evaluation of clinical progress and adherence to prison requirements, as well as blind assessment of risk of violence, dangerousness, and recidivism, as well as criminological factors, by means of international standardized tools including actuarial and structured professional judgment approaches.

Second, discharge locations are the institutions where MDOs are transferred at their release from the forensic clinic (Figure 1). Again, locations are decided by the court, based on the psycho-criminological risk assessment of the MDO’s psychiatric profile and offense behavior management. Release to an institution most often requires a new psychiatric expert assessment to confirm the clinical progress and provide an updated opinion on the prospect of success of the treatment. Three main discharge locations have been identified for Curabilis. If they no longer need external control of their violent behavior, MDOs are transferred to low-security forensic psychiatry wards outside prison for follow-up treatment. If they no longer need intensive inpatient care, MDOs are admitted to a sheltered educational housing and continue their COT in an outpatient setting. Those inmates who have not managed to lower their risk of violence despite intensive treatment, are referred to traditional prison and pursue an outpatient COT while guarantying public safety.

### Data analysis

Age, number of convictions, number of different types of offenses, length of COT at admission, and length of stay were treated as continuous variables. COT-related type of offense, and gender were treated as binary variable. Nationality, education (school drop-out / obligatory schooling / apprenticeship / high school, university), marital status (single / separated-divorced-widowed / married) and children (none / 1 child / 2+ children) were treated as ordinal variables. Psychiatric diagnoses were coded according to ICD 10 codes. Criminal offenses were coded according to the Swiss Penal Code. Cases with multiple diagnoses or offenses were considered in each diagnostic/offense category separately.

Time to failure, time-to-event analysis (Kaplan–Meier survival estimates), were performed to estimate time to release for all participants. Cox proportional hazard regressions were used to predict length of stay, multinomial logistic regressions for nominal variables and logistic regression for binary variables, were used to predict discharge locations at release. All regression models were univariate. For both outcomes, independent predictors were demographic characteristics (age, gender, nationality, level of formal education, marital status, number of children), criminal offenses (type of offense, number of convictions, number of different types of offense), psychiatric diagnosis, and length of COT prior to admission. Comparison between the three main discharge locations was performed by Fisher’s exact test binary variables, Cochran-Armitage test for trend for ordinary variables, and one-way ANOVAs for continuous variables (after Bartlett’s homogeneity of variance testing), or Kruskal-Wallis as nonparametric alternative. The significance level was set at *p* < 0.05. All statistical analyses were performed using Stata 17.0.

## Results

### Descriptive data

Among the 204 participants, 189 (92.6%) were admitted for COT according to Swiss Criminal Code (therapeutic measure, art. 59 SCC) and the remaining 15 (7.4%) MDO were condemned to security measure (indefinite incarceration, art. 64 SCC). Half of the participants have been under COT for more than 18 months prior to their admission to Curabilis, with a wide inter-individual variability from 0 to 15 years (median = 20 months, average 35.8 ± 38.4 [0.0–182.0] months). Nearly all participants (*n* = 186, 91.2%) have been admitted to the forensic psychiatric clinic from regular Swiss prisons, while the few remaining participants came either from general psychiatry wards (*n* = 6, 2.9%) without previous incarceration, or from the acute psychiatric treatment ward located in Curabilis (*n* = 12, 5.9%).

The demographic characteristics of the sample are summarized in Table 1. The typical inmate admitted in Curabilis is a 35-year man slightly more frequently of foreign nationality (57.8%), single and without children. Of importance, about one third of the cases has dropped out from school because of conduct disorders, drug use or violent behavior.

**Table 1:** Demographic characteristics of the sample.

Demographic characteristics	
N	204
Age (years)	35.9 ± 10.7 [19.0–69.0]
Female gender	12 (5.9%)
Nationality	
Swiss	86 (42.2%)
Africa	50 (24.5%)
Western Europe	31 (15.2%)
Asia	16 (7.8%)
Eastern Europe	15 (7.4%)
United States	6 (2.9%)
Education	
School drop-out	63 (30.9%)
Obligatory schooling	72 (35.3%)
Apprenticeship	52 (25.5%)
High school, university	17 (8.3%)
Marital status	
Single	158 (77.5%)
Separated-divorced-widowed	31 (15.2%)
Married	15 (7.3%)
Children (nb)	
None	153 (75.0%)
1 child	25 (12.3%)
2+ children	26 (12.7%)

Table 2 illustrates the type of offenses, and mental disorders of the sample. 78% of the inmates have been convicted for physical violence (bodily harm such as aggression, assault, fight, murder) and 64% for violation of property (such as robbery, organized fraud, or breach of trust). Half of the cases have been convicted for drug-related offenses or violation of domestic privacy (threats, sequestration, and kidnapping). About one third have been convicted for violation against the forces of order, or violation of honor and privacy. Sexual violence and violation of road traffic are reported in 25% of the cases. Deliberately setting fire, illegal immigration and violation of gun law are the less frequent offenses in our sample. 27% of the offenders have committed one single offense. In case of recidivism, half of the participants repeated their offense at least twice with a wide variability up to 22 recidivisms. Multiple types of crimes are found in almost 60% of the cases.

**Table 2:** Offense and mental disease characteristics of the sample.

Criminal offenses (Swiss Penal Code)	
N	204
Single offense	56 (27.5%)
Severity of recidivism (mean; median)	3.6 ± 4.3 [0.0–22.0]; 2.0
Single type of offense	83 (40.7%)
Number of different offenses (mean; median)	4.2 ± 2.0 [1.0–10.0]; 4.0
Type of offense	
Physical violence (art. 111–136)	161 (78.9%)
Property violation (art. 137–172)	131 (64.2%)
Drug trafficking (Lstup, art. 118–123)	108 (52.9%)
Threat, sequestration, kidnapping (art. 180–186)	102 (50.0%)
Violation forces of order (art. 285–295)	77 (37.7%)
Honor and privacy (art. 173–179)	58 (28.4%)
Sexual offense (art. 187–200)	50 (24.5%)
Road traffic laws (LCR, art. 90–99)	48 (23.5%)
Arson (art.221–230)	29 (14.2%)
Illegal immigration (LEtr)	28 (13.7%)
Gun law violation (Larm)	27 (13.2%)
Length of COT (months)	35.8 ± 38.4 [0.0–182.0]; 20.0
Psychiatric diagnosis (ICD-10)	
Schizophrenia, delusional, psychotic disorders (F20-F29)	137 (67.2%)
Psychoactive substance use – SUD (F10-F19)	123 (60.3%)
Antisocial or borderline personalities (F60.2-F60.4)	73 (35.8%)
Intellectual disabilities (F70-F79)	28 (13.7%)
Paranoid or schizoid personalities (F60.0-F60.1)	20 (9.8%)
Mood disorders (F30-39)	15 (7.4%)
Paraphilias (F65)	10 (4.9%)
Developmental disorders (F80-F89)	9 (4.4%)

With respect to the psychiatric diagnoses, psychotic disorders (schizophrenia, delusional disorder) were present in 67% of the cases, 60% suffered from comorbid substance use disorders (SUD), and 35% presented with dramatic, emotional, and erratic personality disorders (Cluster B) such as borderline, narcissistic, or antisocial personalities. Intellectual disability was present in 13% of cases. Less than 10% had a diagnosis of paranoid or schizoid personality disorder. Mood disorders were even rarer. Finally, less than 5% suffered from paraphilias or developmental disorders.

Female gender was significantly associated with a diagnosis of emotional and erratic personality disorders (Male: 66/73, 34.4% vs. Female: 7/73, 58.3%, Fisher’s exact test = 0.0117). There were no other gender-related differences in the prevalence of psychiatric diagnoses.

None of the three main diagnosis, namely psychosis (F20-F29), cluster B personality disorders (F60.2-F60.4) or SUD (F10-F19) were associated with physical violence or psychological violence, such as violations of domestic privacy. There was a clear discrepancy between the relatively high occurrence of sex offenses (24%, *n* = 50) and the low percentage (5%, 10/50) of cases with paraphilia disorders. In the present sample, the most frequent diagnoses in case of sex offenses were SUDs (F10-F19: 30/50, 60%), psychotic disorders (F20-F29: 26/50, 52%) and antisocial or borderline personality disorders (F60-B:24/50, 48%). In parallel, most MDO with sexual crimes were also convicted for physical violence (35/50, 70%), and threat or sequestration (27/50, 54%), indicating that the sexual nature of the crime seems to be associated with a global tendency for violent interpersonal behavior.

To explain the discrepant relationship between sexual offenses and paraphilia disorders, we assessed the association of the former with other psychiatric disorders. As shown in regression models (Table 3), sexual offending was positively associated with antisocial or borderline personality disorders but negatively related to psychotic disorders. There was no effect of SUD on the occurrence of this type of offense.

**Table 3:** Psychiatric diagnosis associated with sexual offense.

Sexual offenses (*n* = 50)			
Predictors	OR	95% CI	Value of *p*
Schizophrenia, delusional, psychotic disorders (F20-F29)	0.42	[0.22, 0.81]	0.010*
Cluster B personality disorders (F60.2-F60.4)	1.98	[1.03, 3.79]	0.040*
SUD (F10-F19)	0.98	[0.51, 1.89]	0.961

*p < 0.05.

### Length of stay and discharge locations

According to the Kaplan–Meier time to release analysis of the 204 inmates, a median stay lasts 2.5 years (31 months). On the lower end, 25% of the inmates stay less 1.5 years, while on the upper end, 25% stay more than 4 years. The 10 shortest stays lasted 2 to 8 months, while the 10 longest stays lasted more than 6 years. The maximum length of stay has been of 7.8 years (94.5 months).

By December 2022, 134 of the 204 inmates were released. Among them, 56% were either transferred into sheltered educational housing (*n* = 44, 32.8%) or open low-security psychiatry wards (*n* = 31, 23.2%). One third of the inmates returned to regular prisons (*n* = 41, 30.6%). About 10 % of the inmates were transferred to their country of origin (*n* = 13, 9.7%) for treatment follow-up in outpatient settings or psychiatric hospitals. Less than 2% were released conditionally without further treatment (*n* = 2, 1.5%). Three participants died during the period of observation (*n* = 3, 2.2%, two suicides).

The lengths of stay were not significantly different between the three main post-release discharge locations (open psychiatric wards / sheltered educational housing / prison), as confirmed by multinomial logistic regression.

### Predictors of length of stay and discharge locations

Among the different types of offenses, drug trafficking, property violation and sex offenses were significantly more frequent among very long (> 4 years) compared to short lengths of stay (< 1.5 years; Table 4A). To predict the length of stay, we ran Cox regression analysis with all our independent predictor variables. Table 4B displays the significant results. A longer length of COT prior to admission significantly predicted a longer length of stay in Curabilis. Three types of offenses emerged as significant predictors of longer length of stay: drug trafficking, violation of property (robbery, organized fraud, or breach of trust) and sex offenses (limit of significance). None of the demographic variables (age, gender, nationality, level of education, marital status, number of children) significantly predicted the length of stay. Diversity of crimes, recidivism, and psychiatric diagnoses were also not related to this outcome.

**Table 4A:** Length of stay quarters-related differences in the sample.

Length of stay	Shortest (< 1.5 years)	Median (1.5–4 years)		Longest (> 4 years)	
Months	0–20	20–31	31–48	48+	Value of *p*
*n* = 116	*n* = 89	*n* = 46	*n* = 40	*n* = 29	
Length of COT (months)	30.1 ± 41.4	39.9 ± 41.0	36.1 ± 37.4	55.1 ± 52.5	0.051
Drug trafficking	38 (42.7%)	30 (65.2%)	18 (45.0%)	22 (75.9%)	0.014*
Property violation	50 (56.2%)	29 (63.0%)	29 (72.5%)	23 (79.3%)	0.010*
Sexual offense	14 (15.7%)	13 (28.3%)	9 (22.5%)	14 (48.3%)	0.002**

* *p* < 0.05, ** *p* < 0.01.

**Table 4B:** Predictors of length of stay using univariate Cox regression models.

Length of stay (N = 204)				
Predictor	Discharge	HR	95% CI	Value of *p*
Length of COT	1.00	0.99	[0.99–0.99]	0.023*
Drug trafficking	1.00	0.64	[0.45–0.90]	0.011*
Property violation	1.00	0.67	[0.47–0.95]	0.028*
Sexual offense	1.00	0.67	[0.46–0.99]	0.050

* *p* < 0.05, ** *p* < 0.01, hazard ratios (HR) <1 indicate longer length of stay.

Table 5A summarizes group differences according to the three main release discharge locations. Cluster B personality disorders and sex offenses were significantly more frequent in MDO returning to prison compared to low-security psychiatric wards. Of importance, Cluster B personality disorders was also less frequent among MDO placed in sheltered education housing than in prison. Conversely, younger MDO as well as those convicted for violation of property were more frequently placed in sheltered education housing compared to prison. There were no significant group differences in respect to the other demographic, psychiatric or criminological data. To predict the discharge location, we ran regression analysis with all our independent predictor variables and displayed significant results in Table 5B. Multinomial logistic regression confirmed that Cluster B personality disorders (antisocial or borderline) were associated with more frequent return to prison. Longer length of COT prior to admission in Curabilis also reduced the chance to be released to low-security psychiatric wards. Compared to the return to prison, sex offenders display a 4.8-fold decrease of their chance of release to low-security psychiatric wards. None of the other demographic, psychiatric or criminological variables predicted discharge location.

**Table 5A:** Subgroups according to main discharge locations.

Subgroups according to main discharge locations (*n* = 116)				
	Prison	Low-security psychiatric wards	Sheltered educational housing	Value of *p*
Predictors	*n* = 41	*n* = 31	*n* = 44	
Length of COT (months)	56.3 ± 68.3	26.9 ± 28.3	36.0 ± 34.1	0.313
Age (years)	40.0 ± 11.7^#^	37.0 ± 10.4	33.1 ± 11.3^#^	0.021*
Cluster B personality disorders	25 (61.0%)^†‡^	7 (22.6%)^‡^	15 (34.1%)^†^	0.003**
Property violation	21 (51.2%)^##^	19 (61.3%)	34 (77.3%)^##^	0.037*
Sexual offense	14 (34.1%)^###^	3 (9.7%)^###^	11 (25.0%)	0.047*

* *p* < 0.05, ** *p* < 0.01, ^#^ *p* = 0.017, ^##^ *p* = 0.014, ^###^ *p* = 0.024, † p = 0.017, ‡ *p* = 0.002.

**Table 5B:** Prediction of main discharge locations using univariate multinomial logistic regression models (cura5 page 23).

Prediction of main discharge locations (*n* = 116)							
Predictors	Prison (*n* = 41)	Low-security psychiatry wards (*n* = 31)			Sheltered educational housing (*n* = 44)		
	RRR	RRR	95% CI	Value of *p*	RRR	95% CI	Value of *p*
Length of COT	1.00	0.98	[0.97–0.99]	0.025*	0.99	[0.98-1.00]	0.095
Age	1.00	0.98	[0.94–1.02]	0.289	0.94	[0.91–0.98]	0.007**
Cluster B personality disorders	1.00	0.17	[0.06–0.47]	0.001**	0.24	[0.09-0-59]	0.002**
Property violation	1.00	1.51	[0.58–3.88]	0.395	3.24	[1.27–8.24]	0.014*
Sexual offense	1.00	0.21	[0.05–0.80]	0.022*	0.64	[0.25-1.64]	0.357

*p < 0.05, **p < 0.01.

## Discussion

Our data provide the first observation of a large sample of MDO admitted for COT in the sole medium-security forensic psychiatric structure for French and Italian speaking inmates in Switzerland. Among the characteristics of this structure, one should note the high densities of prison and mental health professionals working together within the theoretical framework of a forensic therapeutic community, presence of inmates of both genders, and inclusion of a wide diagnostic spectrum. Due to its uniqueness and absence of *a priori* fixed exclusion criteria, the present sample can be considered representative of MDO convicted to COT in the general population.

We will first discuss the demographic, psychiatric and criminological features that characterize the MDO sample of the current study. Second, we will discuss how these determinants predict our two outcome variables, namely length of stay and discharge location.

### Impact of demographic parameters

From a demographic viewpoint, three main findings merit further consideration. First, our data confirm the scarcity of women among MDO under COT as recently reported by Tomlin et al. (35) in their study of MDO placed in forensic facilities in 17 European countries. The lowest percentage of women under COT was reported in Slovenia (5%) and the highest in England and Wales (18%). However, in this latter case, the authors included sentenced prisoners transferred to forensic hospital units but also patients detained for treatment under civil mental health law. In comparison, the percentage of women in our sample is of 5.9%. Another Swiss study reported 7.9% of female offenders without distinction between regular inmates and COT (36). The rate of women in the present study is also below the 8.6% reported in Austria for MDO with abolished criminal responsibility (17). In contrast to male MDO, who tend to commit more violent and narcotics-related crimes (28), the lower prevalence of female MDO in forensic inpatients settings could reflect lower levels of comorbid SUD and higher use of psychiatric inpatient treatment in general psychiatry rather than specialized forensic settings. In our sample, Cluster B personality disorders were more frequent among women, yet there was no gender-related difference in the prevalence of SUD. These results confirm the interest of including female participants and the need for further studies on gender-specific differences in MDO with personality disorders.

Second, the association between single marital status and COT in this study is in line with previous observations regarding the protective role of marriage against offending in psychiatric patients. One recent study reported that single status is a strong risk factor for criminal recidivism in community settings (37). In the same line, the analysis of offenders with COT in Denmark during 1980–1992 revealed that they are predominantly single compared to regular prison inmates (38). Third, contrary to our hypothesis, age emerges as a significant predictor of discharge location. Younger age is associated with increased chance to be released from prison and transferred to sheltered educational housing in our study. This observation is consistent with the better adherence to rehabilitation programs reported for younger inmates with higher motivation to change (39). It raises the interest of having age-specific wards in forensic psychiatric clinics.

### Association between clinical diagnosis and criminological characteristics

Clinically, MDO in Curabilis have mainly been convicted for physical violence, threat, kidnapping, and violation of property, as well as drug-related offenses. The most prevalent mental disorders were schizophrenia and delusional disorders, with a marked comorbidity of SUDs, followed by antisocial and borderline personality disorders, and intellectual disability.

Consistent with a previous study (40), most of our patients have been diagnosed with co-occurring mental and SUDs, but only a low percentage met the criteria for antisocial personality disorder. MDO represent a clinically distinct group with an overrepresentation of psychotic disorders compared to regular inmates with mental disorders as already reported in several previous studies (4, 9, 18, 41–44). A modest but consistent association between interpersonal violence and schizophrenia combined with comorbid SUD has been reported over the last decades (45, 46). Violence toward others is found in psychotic patients with comorbid traits of psychopathy, and in first-episode untreated individuals, who suffer from acute symptoms of psychosis and may have aggressive behaviors because of the impact of their positive symptoms (47, 48). The presence of comorbid SUD steadily increased the risk of violence among schizophrenics (form 3-fold to 10-fold increase compared to the general population) (49). Contrasting with previous reports on the link between violent crime, and schizophrenia, personality disorders and SUD (49, 50), such associations were not found in the present sample. One possible explanation for this discrepancy could be that the decision of COT for schizophrenics in Switzerland is not related to the severity of the offense, since its main objective is the prevention of criminal escalation in patients with long-standing psychiatric vulnerability.

While psychotic illnesses such as schizophrenia represent a main diagnosis in most inpatient care forensic services, the place of personality disorders in mental disorder defense is by far more ambiguous (1). For instance, personality disorders were not considered in this context in France. In contrast, nearly 37% of the patients in forensic psychiatric hospitals in Germany have a primary diagnosis of personality disorders (6). Personality disorders were the second most prevalent diagnoses (34%) in high security patients with mandatory placement in forensic psychiatric centers in Belgium (18, 51). Johnson and Elbogen (51) postulated that the high incidence of this pathology in criminal populations, as well as the difficulty to determine direct causality between their presence and criminal act, and define the cut-off between traits and clinically overt disorders, may explain the variability of prevalence rates for this condition among MDO. Our results are consistent with the prevalence rates reported in Germany and Belgium, pointing to the progressive change of the clinical conception of these disorders that are no longer considered under volitional control. Importantly, Jeandarme et al. (18) suggest that in countries admitting decreased or abolished responsibility, psychiatrist-judicial experts are more likely to conclude that patients with personality disorders are unable to control their behavior. Some authors have suggested that violent individuals with schizophrenia and antisocial personality disorder share common emotion processing deficits such as facial affect recognition, which might benefit from transdiagnostic treatment targets (52).

Sex offenders represented 25% of the criminal offenses in our study, but paraphilias were found only in 5% of the sample. For some authors, sex offenses are frequently associated with psychotic symptoms (53) but may also be the consequence of criminogenic motivations, especially substance use and paraphilic interests (54). The role of serious mental illness among those who sexually offend is still matter of debate. Recently, some authors have shown that sex offenders did not differ significantly in their demographics, psychiatric diagnoses, or recidivism risk compared to non-sex offenders found not guilty for reason of insanity (55). Interestingly, in our study, psychotic disorders decreased the risk of sex offenses, while Cluster B personality disorders steadily increased this risk. In contrast, SUDs had no effect on the sexual nature of offenses. Moreover, most MDO convicted for sexual crimes were concurrently committed for physical violence and threat or sequestration. The sexual nature of the crime seems thus to be secondary to an overall tendency for violent interpersonal behavior. In France, COT was originally implemented in 1998 for people convicted of sex offenses only, before being extended to other serious non-sexual crimes in the 2000s (19). A German study has found the same prevalence of mental disorders in sex offenders and non-sex violent offenders in regular prisons, but also in sex offenders in forensic psychiatry. In agreement with our data, SUDs were the most frequent diagnosis in the three groups, yet the prevalence of comorbid personality disorders was significantly higher in the sex offenders in forensic psychiatry (85%) compared to the two other groups (56).

### Determinants of length of stay

Duration of inpatient forensic psychiatry care for COT is an essential issue, because of the high costs of medium and high-security hospitals in prison. In our sample, most cases followed outpatient COT in regular prisons for a median of 2 years prior to their admission to Curabilis with a large variety from 0 to 15 years. The median stay in our medium-security prison-based inpatient service lasts 2.5 years, with a range from 0 to 8 years.

Tomlin et al. (35) examined length of stay for forensic inpatients in 12 European countries and reported that the curve of the mean length follows a bipolar distribution. Seven countries exhibit a mean length under 3.5 years with the remaining states averaging over 7 years. The variability among countries is high, ranging from 1 to 10 years. In their worldwide review, Beis et al. (1) confirmed that the duration of forensic hospitalization is mainly influenced by the variations of the country’s legal frameworks and cultures, and the management of MDO is an indicator of the country’s ability to maintain public safety and preserve basic human rights. In England, long-stay is defined as 5+ continuous years in medium secure care or 10+ years in high secure care or a combination of the high and medium secure settings totaling 15+ years (22). Hospitals in Netherlands must apply for escorted leave for every patient within 1 year of admission, unescorted leave with 4 years and transmural leave within 6 years enabling patients to move through the system quicker (2). Countries like Austria and Switzerland define no legal time limits to COT for MDOs, the length of stay being dependent on the clinical evolution (17).

Several studies addressed the baseline determinants of length of stay in long stay forensic services with conflicting data. Severity of index offense, sex offense, high crime recidivism, psychotic disorder (persistent psychosis), treatment resistance, duration of mental illness, history of SUD, previous contacts with child and adolescent psychiatric services, as well as older age have been related to longer length of stay (29–33). In addition, external factors related to the judicial system, criteria for admission, and allocation of resources may also impact on this parameter (1). Our findings indicate that length of stay and location at release are independent. Importantly, neither demographic variables, nor severity of crime recidivism and psychiatric diagnosis at admission predicted treatment duration. Length of COT prior to admission, drug trafficking and violation of property as well as sex offenses were the only factors to be significantly associated with increased length of stay, pointing to the fact that past criminological factors are more relevant than baseline clinical characteristics for predicting length of stay in medium-security hospitals for COT in Switzerland. While sexual offenses and length of COT impacted on treatment duration, personality disorders were not related to the later. This finding does not support the idea of a relationship between debilitating psychiatric conditions and longer length of stay. MDO with COT in Switzerland are admitted and released by court decision and detained under legal order, based on the level of dangerousness, as clearly illustrated by these offense-length of stay association. Our findings are consistent with a comparison in forensic patients with and without a restriction order of unvoluntary treatment realized in Sweden, which has shown that involuntary treatments are related to convictions for violent crime, but not to any other differences in demographic or clinical variables (30).

### Predictors of discharge locations

In the present study, the large majority (more than 90%) of the MDO were admitted to the forensic psychiatric clinic from regular prisons. At release, more than half (56%) of the admitted inmates successfully managed to lower their risk or recidivism enough for the court to deliver a verdict of prison release. Younger age and past conviction for property violation (rather than physical violence) increased the chances to be admitted to sheltered housing. A Swedish study comparing MDO sentenced to prison vs. compulsory forensic psychiatric treatment after severe violent or sexual crimes showed that the later individuals spent significantly more time at liberty after discharge and had fewer relapses compared to the first (57). Our findings add evidence to previous reviews on European forensic psychiatry (4), and confirm our hypothesis that forensic-psychiatric care may produce better outcome than incarceration in prison alone. Interestingly, MDO with longer COT prior to admission have significantly less chance to be released from prison, stressing the need for providing specialized forensic care as soon as possible after the conviction to avoid the deleterious effect of long-term incarceration (4).

One main finding of the present study is that the risk to return to prison was significantly higher in MDO with antisocial and borderline personality disorders. This finding confirms our hypothesis, and parallels previous evidence showing that the presence of this type of personality disorders is associated with 4.7-fold increase of the risk of a new incarceration among regular inmates discharged into the community (58). Whether or not personality disorders can be treated in secure settings remains a matter of debate. Among this group of COT inpatients, it has been suggested to distinguish those who respond to long-term therapeutic approaches within the scope of reduced risk of recidivism from those who are treatment-resistant with poor prognosis and persistent risk of reoffending (18). A dimensional model of personality, such as defined by the newly published ICD-11 manual, could provide additional cues to define the severity of the personality disorder, and its relationship to offending, rehabilitative prospects and community protection (59).

Finally, our findings showed that MDO with sex offenses more frequently return to prison at the end of their stay in Curabilis. They confirm that MDO with more severe criminal background have the lowest chances to be retained in imprisonment. Previous evidence concluded that the outcome of specific sex offender treatment programs remains disappointing (4). As in our study, while not all sex offenders have a mental disorder, up to half have been diagnosed with a comorbid personality disorder (60, 61). Sex offender treatment non completion has been related to the diagnosis of personality disorder or psychosis (61). It is thus likely that sexual violence is a main adverse factor that drastically decreases the chance of freedom of inmates with Cluster B personality disorders implying an increased need for psychiatric care in a secured prison environment.

### Strengths and limitations

Strengths of the present study include the assessment of a large homogeneous sample of MDO in the only Swiss forensic psychiatry clinic for the French and Italian speaking parts of Switzerland. All 204 participants were submitted to the same COT based on therapeutic community approaches. Further, psychiatric diagnoses were defined using standardized ICD-10 criteria, assessed by an independent psychiatry expert as part of the court ordered investigation prior to admission into the forensic clinic.

Several limitations need to be considered. First, the present sample concerned inpatient COT treatment, yet a significant proportion of offenders receive outpatient COT interventions in regular prisons. Subsequently, the present findings are not necessarily valid when the full spectrum of COT is considered. Second, the clinical diagnosis is often modified after the observations made during the hospital stay. Keeping as the gold standard the diagnosis of the expertise during prosecution introduces a bias that should be considered when interpreting our data. Third, and to be close to real life, the diagnosis of personality disorders was made without standardized questionnaires by psychiatric experts. Fourth, length of stay and treatment pathways in COT are impacted by several factors such as presence of family support, working skills, psychiatric history as well as severity and duration of mental illness. These parameters were not considered in the present analysis that focuses on criminal characteristics and clinical diagnosis, treated as binary variables. Finally, this study has no medico-economic arm so that we cannot comment on the cost/effectiveness of the care programs in Curabilis in particular for inmates with personality disorders and/or sex offenses. Future studies in larger samples addressing these limitations are needed to identify the predictors of clinical trajectories and define MDO subgroups that can optimally benefit from COT.

## Conclusion

This first exploratory study on COT for MDO in Geneva provides an introductory insight into the complexity of the treatment pathways for a socially sensitive and ethically challenging group of inpatients. It makes it possible to define the main determinants of length of stay and outcome considering clinical and criminological variables.

These observations are relevant from a medico-economic viewpoint. The complexity of care programs in Curabilis, based on the interaction between health and prison professionals, leads to high costs, superior from those of care in regular prison or psychiatric hospitals. It is thus necessary to reserve this approach to carefully selected MDO subgroups. The present data suggest that inmates (and especially sex offenders) with borderline and antisocial personality should be carefully screened before and after admission for their adherence to care programs and clinical evolution to avoid long and inefficient stays.

## Data availability statement

The original contributions presented in the study are included in the article/supplementary material, further inquiries can be directed to the corresponding author.

## Ethics statement

The studies involving humans were approved by Swiss Association of Research Ethics Committees 2022–00739. The studies were conducted in accordance with the local legislation and institutional requirements. Written informed consent for participation in this study was provided by the participants’ legal guardians/next of kin.

## Author contributions

KW, CM, PB, and PG contributed to the conception and design of the study. KW and PG wrote the paper. SM and LL extracted the data. KW supervised the database. FH performed the statistical analyses. All authors contributed to the article and approved the submitted version.

## Footnotes

^1^An English version of the Swiss Criminal Code is available here: https://www.fedlex.admin.ch/eli/cc/54/757_781_799/en
